# Climate change decreases suitable areas for rapeseed cultivation in Europe but provides new opportunities for white mustard as an alternative oilseed for biofuel production

**DOI:** 10.1371/journal.pone.0207124

**Published:** 2018-11-05

**Authors:** Rafael Jaime, Julio M. Alcántara, Antonio J. Manzaneda, Pedro J. Rey

**Affiliations:** 1 Departamento de Biología Animal, Biología Vegetal y Ecología, Universidad de Jaén, Jaén, Andalucía, Spain; 2 Centro de Estudios Avanzados en Energía y Medio Ambiente (CEAEMA), Universidad de Jaén, Jaén, Andalucía, Spain; Fred Hutchinson Cancer Research Center, UNITED STATES

## Abstract

Oilseed crops, including several mustards, are cultivated as biofuel sources worldwide. However, common mustard crops (e.g., the rapeseed *Brassica napus*) grow naturally in mesic temperate regions, which are expected to be impaired by global warming and increased aridity. In particular, increased aridity is predicted to reduce the oil concentration and seed yield of rapeseed crops. There is therefore an urgent need to identify alternative bioenergy crops that are preadapted to future climatic conditions. An alternative to conventional *Brassica* species for biodiesel production is the white mustard *Sinapis alba*, which is native to the circum-Mediterranean region and has a high seed lipid content. *S*. *alba* grows spontaneously in olive groves and other widespread Mediterranean crops; accordingly, it could be easily cultivated by companion planting to improve ecosystem function by decreasing soil loss, controlling microbial disease, and assisting in the maintenance of biodiversity. In this study, using species distribution modeling, we predicted climatically suitable areas for the cultivation of *S*. *alba* in Western Europe across the Mediterranean Basin under present climatic conditions and several climate change scenarios. We show that current climatically suitable areas for *S*. *alba* cultivation do not overlap with those for *B*. *napus*. Unlike *B*. *napus*, *S*. *alba* could be cultivated throughout most of the circum-Mediterranean region. According to our models, increases in aridity and annual mean temperatures will expand the climatically suitable areas for *S*. *alba* in the Mediterranean Basin. However, suitable areas for the cultivation of *B*. *napus* will decrease significantly in Western Europe. Our results indicate that *S*. *alba* is a strong, environmentally safe candidate for biofuel production throughout the Mediterranean Basin and other Western European countries, especially under climate change scenarios that are expected to impair current oilseed crops.

## Introduction

The effects and consequences of climate change are increasingly palpable. Many studies have shown and/or predicted the adverse effects of climate change and global warming causes in different environments [1–3]. The impact of the rapid rise in temperature across the Earth’s surface is already discernible in many animal and plant populations [4]. In particular, the effects of global warming on economically important crops are a serious concern [5–12].

Global warming has increased interest in potential sources of biofuels in the last decade [13] as alternatives to petroleum-derived fuels (i.e., [14–15]). The development and production of biofuels is a particularly important issue in states with a strong dependence on petroleum imports. Biofuels are mainly produced from energy crops; biodiesel is produced from oilseed species, such as soybean (*Glycine max*, Fabaceae), rapeseed (*Brassica napus*, *B*. *rapa*, and *B*. *juncea*), sunflower (*Helianthus annuus*, Asteraceae), flax (*Linum usitatissimum*, Linaceae), and the African oil palm (*Elaeis guineensis*, Palmaceae) [16]. The cultivation of these species, however, has important environmental costs (see [16–21]).

Oilseed crops, like other crops, are facing the effects of climate change. In particular, temperature increase and precipitation reduction are important climatic factors determining yield and seed oil concentration [7, 22–24]. There is therefore a need to investigate alternative energy crops preadapted to future temperatures and precipitation levels predicted by climate change models. Despite studies of the production of biodiesel and other biofuels from alternative species, especially in the agronomic and technological fields (i.e., [18, 25–26]), the ecological requirements for these cultivars and adaptive variation in seed and ecophysiological traits are unclear [27–28]. Ecological requirements for plant species, under the concept of the ecological niche, are used to identify suitable current and future areas for cultivation (i.e., [29]). Analytical tools developed in ecology to model species niches and potential distributions [30–33] enable the projection of changes in the distribution of climatically suitable areas for the cultivation of these energy crops under different scenarios of climate change [34–35].

Brassicaceae play a prominent role among the plant families with high potential for biofuel production from oilseeds [25, 36–40]. Brassicaceae species, especially within *Brassica* genus, are used for biofuel production in Europe and North America. Varieties of *Brassica napus* (rapeseed), *B*. *rapa*, and *B*. *juncea* are major sources of biofuel [41]. Rapeseed is an annual herb from relatively cool and humid temperate climates. In 2008 in Europe, rapeseed accounted for 79% of all feedstock crops used for biodiesel production [40, 42]. Global warming and associated increases in aridity in many temperate regions where they are currently cultivated may nonetheless compromise crop production. To meet future oilseed demand, a potential approach is the development of new alternative crops of oilseed mustards that are naturally adapted to more xeric conditions. Mediterranean climate regions of the world are hardly adequate for the optimum cultivation of rapeseed [43]. However, many other species of *Brassica* and *Sinapis* are frequently found in Mediterranean oldfields, some of which produce seeds with a high lipid content [25, 44]. In particular, *Sinapis alba* (hereafter white mustard) is a candidate for the production of biodiesel and an additive lubricant for diesel [25–26, 39]. However, potentially suitable areas for the cultivation of white mustard are unknown; thus the extent to which white mustard could complement the current and future areas of rapeseed cultivation is unknown. This is important since global warming will impact the distribution and suitability of other economically important crops (i.e., [24, 29, 43, 45]).

In this study, we model the suitable potential area for white mustard in the Mediterranean Basin under current and future climatic conditions using different general circulation models (GCMs) and emission scenarios. We also explore the suitable areas for rapeseed cultivation according to a crop model made using Global Agro-Ecological Zones (a FAO resource for crop suitability distributions; GAEZ hereafter [46]). We address the following questions. (1) Do currently suitable areas for rapeseed and white mustard overlap, making them potentially alternative crops in the same zone, or are they complementary at the continental level? (2) Will the distribution of suitable areas for these species expand or retract in Europe during the 21st century under different climate change scenarios? (3) Given the projected suitable areas for the cultivation of these species, should European countries focus on the research and development of white mustard oilseeds as an alternative to rapeseed for biofuel production in Europe?

## Materials and methods

### Study species

*Sinapis alba* (Brassicaceae), commonly known as white mustard, is an annual plant native to the Mediterranean Basin but that can now be considered cosmopolitan owing to its wide cultivation. It can reach 100 cm tall, with erect branched stems, usually with stiff trichomes. The fruit is a silique 3–6.5 cm long containing 2–5 seeds of 2–3.5 mm in diameter [47]. It is considered a weed and occupies road ditches, derelict sites, but also vineyards, olive groves, and other crop fields. It prefers calcareous and nitrified soils [48]. White mustard is typically cultivated for white mustard seed production [44] and is a potential source of biofuel owing to the high lipid content of its seeds [25–26, 44]. The natural fat content of its seeds ranges from 25% to 41% [25–26, 44]. It is traditionally used for pharmacological, fungicide, or culinary purposes [37, 49].

*Brassica napus* (Brassicaceae), commonly known as rapeseed, is an annual or biennial plant from Central and Southern Europe and Western Asia. It requires at least 400 mm of annual rainfall. It tolerates winter droughts well and, in the rosette state, is able to withstand very cold temperatures. It requires deep and well-drained soils. It reaches 150 cm tall, with erect branched stems, usually with stiff trichomes. The fruit is a silique containing 12–18 seeds of 1.1–1.8 mm in diameter. Rapeseed is typically cultivated for the production of animal feeds, edible vegetable oils, and biodiesel [45]. The fat content of its seeds cultivated ranges from 38% to 45% [44].

### Modeling the white mustard distribution

The analyses and results for white mustard reflect topo-climatic suitability (i.e., only climatic and topographic variables were considered), but the data for rapeseed reflect agro-ecological suitability (i.e., environmental and agronomical data were incorporated). Unless otherwise stated, for simplicity, the general term suitability is used in both cases. To determine geo-climatically suitable areas for white mustard growth in the Mediterranean Basin (ranging between 50.46° N and 26.91° N and -19.04° W and 44.47° W) under present and future conditions (2050 and 2080), species distribution modeling (SDM) was performed based on presence/absence data and environmental variables from the same localities.

#### Presence/Absence data

The distribution of *S*. *alba* was modeled using presence/absence data, which allows fitting and model evaluation without assuming pseudo-absences. Presence/absence data improve model performance over presence-only data [50, 51]. However, presence data for cultivated species in global databases are likely to contain many false presences in localities where the species is cultivated but would not be able to persist under natural climatic or edaphic conditions. Thus, during the late spring and early summer of 2013 and 2014, *in situ* geo-referenced presence/absence field data were obtained throughout the natural range of the species in the southern Iberian Peninsula [47]. A total of 15 routes were surveyed, spanning approximately 3000 km. Systematic stops were made along each route at intervals of 3–10 km (typically 5 km), depending on the route characteristics (topography, land use, and type of roads), and the presence/absence of the species was registered. In total, 174 presences and 347 absences of the species were obtained (geographic coordinates are available online in supplementary material S1 Table). The field monitoring did not involve collecting any plant material, and therefore, no specific permissions were required for visiting the studied locations. The field studies did not involve endangered or protected species.

#### Environmental variables

The SDM included 22 variables. Environmental rasters of 30 arc-second resolution were collected for 19 climatic variables and altitude from the WorldClim version 1.4 ([52]; http://www.worldclim.org/), and potential evapotranspiration (PET) was obtained from http://www.cgiar-csi.org/. The slope was derived from the altitude layer using QGIS 2.8.1. Soil data were not included because the information available for most countries in which the intention was to project the SDM (i.e., North African, Southwest Asian, and non-EU countries) did not have sufficient resolution to be useful in SDM at the scale considered in this study (i.e., the best global soil database available to date, the Harmonized World Soil Database v 1.2 from FAO, has a 5 arc-minute resolution).

The environmental layers were masked to span the Mediterranean Basin using ArcMap ArcGIS 9.3 (ESRI, Redlands, CA, USA). To avoid overparameterization, the climatic variables and PET were reduced by a varimax-rotated principal components analysis (PCA). The PCA identified four factors with eigenvalues exceeding one, explaining 86.14% of the total variance. The first factor was influenced mainly by annual mean temperature (Bio1) (loading factor = 0.95). The second axis was influenced by the maximum temperature of the warmest month (Bio5) (-0.96). The third axis was influenced by annual precipitation (Bio12) (-0.98). The last axis was influenced by isothermality (Bio3) (0.97). Based on the high factor loading for the main variable explaining each PC, these four climatic variables were retained for the SDM.

Among the six environmental variables (Bio1, Bio3, Bio12, Bio5, Altitude, and Slope), a strong correlation (*r* = -0.94; p < 0.05) was found between Bio1 and altitude; accordingly, altitude was removed from the modeling.

#### Fitting the SDM

The 521 localities were divided into training and validation datasets. The training dataset consisted of 2/3 of the localities extracted randomly using ArcMap ArcGIS 9.3, and the remaining localities served as validation data. The SDM was fitted to the training dataset by generalized linear models (hereafter GLM) [53] with a binary response variable (presence/absence) and logit link-function, using Pearson’s chi-square to control for overdispersion, in Wolfram Mathematica 10.0. The GLM was a 2^nd^ degree polynomial regression incorporating the linear and quadratic terms of the climatic variables and the slope of the terrain. Compared with more complex modeling techniques (e.g., neural networks, generalized additive models, random forests, or multivariate adaptive regression splines; [54–55]), models with low complexity (such as 2^nd^ degree polynomial regression models) tend to provide a lower fit to the training data but tend to have better transferability in space [56] and likely in time ([57]; but see [58]); accordingly, they are better suited for the objectives of the present study. To assess model performance and allow comparisons with other studies, following [59], the validation dataset was used to calculate two performance statistics: TSS (true skills statistic) and AUC (area under the receiver-operating characteristic curve). TSS considers both omission and commission errors and success as a result of random guessing; it ranges from -1 to 1, where 1 indicates perfect agreement and values of zero or less indicate a performance no better than random [59]. AUC represents the expected proportion of positives ranked before a uniformly drawn random negative; it varies from 0 to 1, whereby a model whose predictions are 100% incorrect has an AUC of 0 and one whose predictions are 100% correct has an AUC of 1 [56, 60–61].

**Fig 1:**
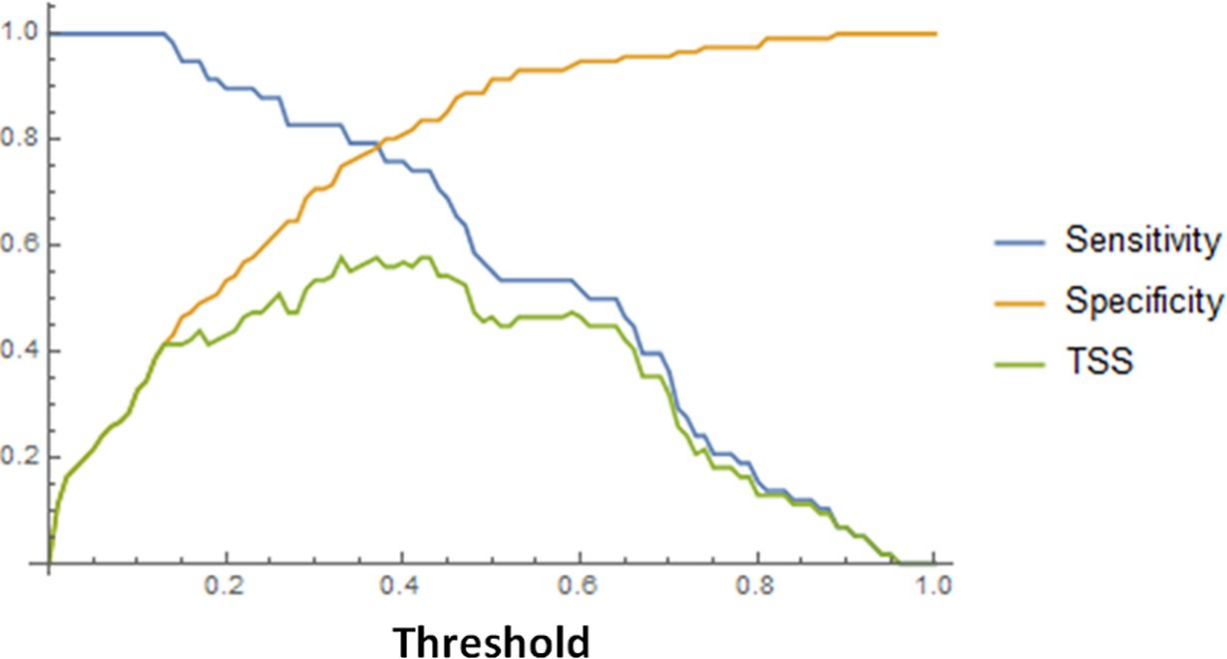
Sensitivity, specificity, and TSS plotted against threshold values. The optimal threshold was defined at the point at which the sensitivity and specificity curves crossed (threshold: 0.37). TSS = 0.578 for the validation sample.

To interpret model results, it is important to identify a threshold value above which model outputs accurately indicate the presence of a species [62]. There are many ways to define this threshold. Sensitivity (true positive predictions vs. the number of actual positive sites) and specificity (true negative predictions vs. the number of actual negative sites) were plotted against a series of thresholds, and the threshold value was selected as the point at which the two curves crossed (Fig 1, threshold: 0.37; [63] in [62]). This procedure balances the cost of an incorrect decision and the benefit gained from a correct prediction [64]. Finally, the SDM model was validated by logistic regression of the observed presence/absence against the predicted probability of occurrence using the validation sample.

#### Future projections

We projected the fitted SDM for the climatic conditions forecasted for 2050 and 2080 under two greenhouse gas emission scenarios. RCP4.5 assumes an intermediate impact on climate change and is characterized by stabilization without overshoot to 4.5 W/m^2^ at stabilization after 2100. RCP8.5 has a severe impact on climate change and is characterized by a rising radiative forcing pathway leading to 8.5 W/m^2^ in 2100 [65]. These scenarios were applied using the outputs from five different GCMs: ACCESS1-0 [66], CCSM4 [67], HadGEM2-CC [68], MPI-ESM-LR [69], and MRI-CGCM3 [70]. These GCMs were downscaled and calibrated (bias corrected) using the WorldClim 1.4 climate as a baseline ‘current’ climate. For each date and emission scenario, we ensembled the projections obtained using the five GCMs. The projections were prepared into binary 1/0 (presence/absence) layers applying the model threshold. Then, the five thresholded projections were summed to obtain an ensemble map with pixel values ranging from 0 to 5 according to the number of GCMs that predict the presence of the species. Using this procedure, four maps were obtained, representing the combinations of two years (2050 and 2080) and two greenhouse gas emission scenarios (RCP4.5 and RCP8.5).

### Projections of crop suitability for rapeseed

GAEZ (http://www.gaez.iiasa.ac.at/) was used to generate rapeseed models of the crop suitability index under current 2050 and 2080 climatic projections in Europe, Russia, North Africa, and the Near East. GAEZ is a very useful and contrasted tool that provides information about crop suitability, enabling the development of reliable models of the suitable area for rapeseed. GAEZ makes projections over a geopolitical spatial area based on country boundaries, which resulted in a projection area different from that used for white mustard, and this difference must be considered when comparing the predictions for each species. Climatic input variables for the model were average temperature, diurnal temperature range, sunshine fraction, wind speed at 10 m, relative humidity, wet‐day frequency, and precipitation. Modeling generation options in GAEZ were set as follows: rain-fed water supply, high-level inputs/advanced management, CO_2_ fertilization, and three different GCMs (CSIRO Mk2, Hadley CM3, and MPI ECHAM4) with two different greenhouse gas emission scenarios (A2 and B2). These GCMs were different from those used in the modeling of *S*. *alba*, since the GAEZ procedure only offers a few GCMs options, from CMIP3, and these are no longer available. It was necessary to use similar GCMs from CMIP5. It should be noted that the predictions of CMIP3 have been overcome; accordingly, the lack of suitable area in the case of rapeseed should be interpreted as a conservative estimate. Similarly, climate change scenarios that were similar to RCP8.5 and RCP4.5 were used. According to Maloney et al. [71] the CMIP5 scenarios RCP8.5 and RCP4.5 are comparable to the A2 and B2 scenarios of CMIP3.

For each year and greenhouse gas emission scenario, the forecast maps generated by GAEZ under the three GCMs were ensembled. The ensembles constructed in this case differ from the procedure we used to build the ensembles in the SDM of white mustard. First, we applied a threshold crop suitability index of 0.8 to the current and forecast maps. Then, the pixel values of the ensemble were recalculated on a scale from -3 to 3, where negative values indicate the number of GCMs predicting that a currently suitable pixel will become unsuitable for the species, 0 indicates that the species is not present currently or in the forecasted future according to all GCMs, and positive values indicate the number of GCMs predicting that a currently suitable pixel will remain suitable for the cultivation of the species. Using this procedure, four maps representing the combinations of two years (2050 and 2080) and two greenhouse gas emission scenarios (A2 and B2) were obtained.

## Results

### White mustard distribution model

The fitted model had a TSS of 0.578 (Fig 1) for the validation sample. This value was high compared with previously reported values [60] for 638 European plant species, especially when compared with other annual and ruderal species, since models for these species tend to exhibit relatively poor performance. The AUC was 0.863, which is relatively high compared with the values reported for many other plant species [56, 60–61]. The proportion of deviance explained by the model (*D*^2^) was 27.68%, with an Efron pseudo-*R*^2^ of 0.3267. The fitted model showed that the probability of occurrence of white mustard maintained a hump-shaped relationship with the maximum temperature of the warmest month (Bio5, negative quadratic term) and slope (Slope, negative quadratic term) (Table 1), what suggest that there exists an optimum of these variables for plant occurrence. At the threshold value (0.37, Fig 1), the model specificity and sensitivity were 0.78. The logistic regression of the observed presence/absence in the validation sample on the predicted probability of occurrence showed a statistically significant fit (*y* = -3.042 + 6.124*x*; SE = 0.90; z = 6.83; P = 8.52071 × 10^−12^) with an explained variance of 0.3669, as estimated by Efron pseudo-*R*^2^.

**Table 1:** Summary of the GLM fitted to presence/absence data for white mustard in the Mediterranean Basin. The independent variables are as follows: Bio1, Annual mean temperature; Bio3, Isothermality; Bio5, Maximum temperature of the warmest month; Bio12, Annual precipitation; and Slope.

Variable	Estimate	Standard error	z-statistic	P-value	DF	Deviance	Residual DF	Residual deviance
	-274.528	109.707	-2.502	0.012			346	435.004
**Bio1**	0.269	0.318	0.846	0.397	1	4.551	345	430.453
**Bio3**	4.095	5.134	0.798	0.425	1	49.013	344	381.441
**Bio5**	**1.091**	**0.355**	**3.074**	**0.002**	1	25.330	343	356.111
**Bio12**	-0.015	0.021	-0.701	0.483	1	4.057	342	352.055
**Slope**	**0.623**	**0.166**	**3.750**	**0.000**	1	16.418	341	335.636
**(Bio1)**^**2**^	-0.000	0.000	-0.636	0.525	1	5.0597	340	330.577
**(Bio3)**^**2**^	-0.062	0.069	-0.901	0.368	1	0.784	339	329.793
**(Bio5)**^**2**^	**-0.001**	**0.000**	**-3.002**	**0.002**	1	10.187	338	319.605
**(Bio12)**^**2**^	0.000	0.000	0.569	0.569	1	0.224	337	319.382
**(Slope)**^**2**^	**-0.035**	**0.016**	**-2.145**	**0.032**	1	4.798	336	314.583

Significant effects at P < 0.05 are in bold-type.

**Fig 2:**
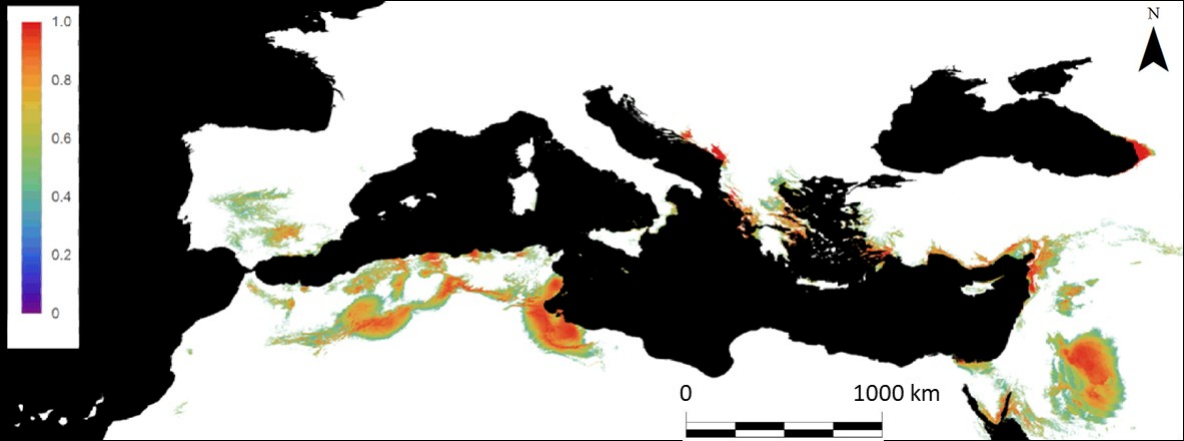
Current suitability map of white mustard in the Mediterranean Basin based on the GLM results. Only pixels with suitability values equal to or greater than the 0.37 threshold are shown.

**Fig 3:**
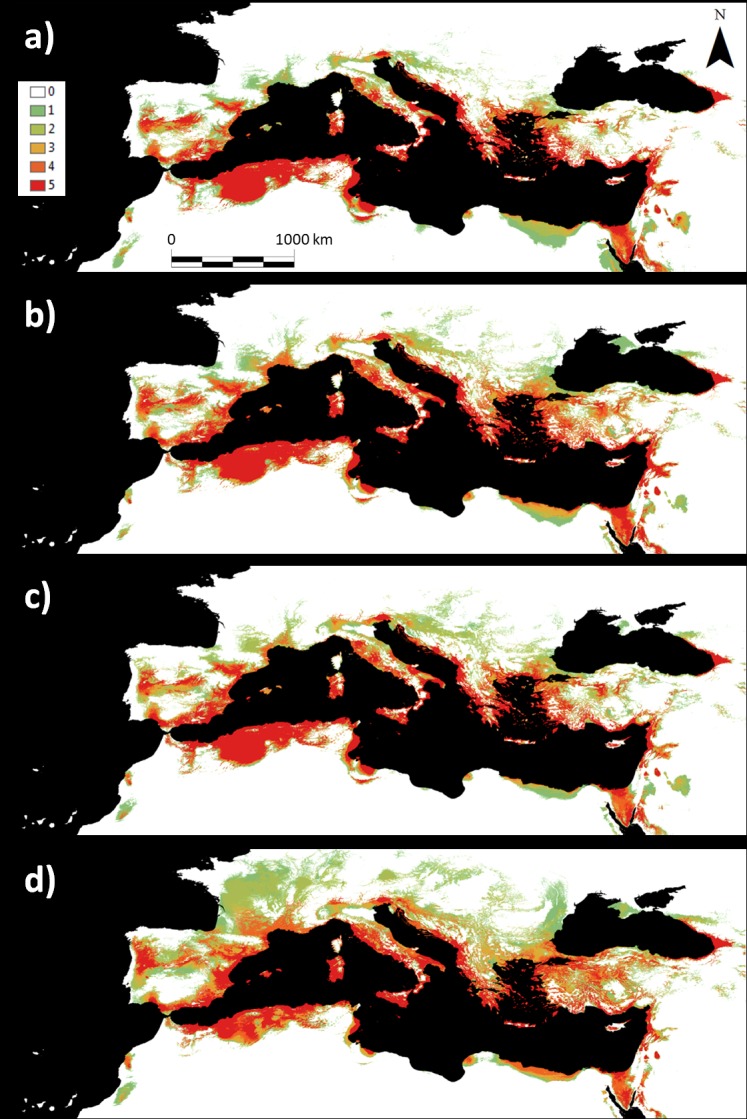
Ensembled projections of the fitted GLM for white mustard suitability using five GCMs. a) 2050 and RCP4.5 emission scenario, b) 2050 and RCP8.5 emission scenario, c) 2080 and RCP4.5 emission scenario, and d) 2080 and RCP8.5 emission scenario. The color scale represents the number of GCMs that predicted the presence of the species for each combination of year and greenhouse gas emissions scenario.

The currently suitable area for the species (probability values above the 0.37 threshold in the model) (Fig 2) spanned 56691.3 × 10^3^ ha, representing 8.5% of the total projection area. The four ensemble maps indicate that the suitable area for white mustard cultivation would increase over time and spread throughout the Mediterranean Basin (Fig 3). According to our model, the suitable area would increase in 2050 to between 62044.3 × 10^3^ ha and 123552.1 × 10^3^ ha (depending on the GCM and emissions scenario) and between 65914.8 × 10^3^ ha and 136008 × 10^3^ ha in 2080 (Table 2). The increases in suitable areas were between 9.4% and 117.9% in 2050 and 16.3% and 139.9% in 2080.

**Table 2:** Extent of topo-climatically suitable land for white mustard growth for different GCMs and greenhouse gas emission scenarios in 2050 and 2080.

General circulation model	Greenhouse gas emission scenario	Suitable land in 2050	% of projection area	Area lost in 2050 relative to current projected area	% of area lost in 2050 relative to current projected area	Suitable land in 2080	% of projection area	Area lost in 2080 relative to current projected area	% of area lost in 2080 relative to current projected area
**ACCESS1-0**	RCP4.5	94923.8	14.3	29184.3	4.4	103864.9	15.7	35279.0	5.3
	RCP8.5	112813.7	17.0	35384.1	5.3	127907.6	19.3	43878.0	6.6
**CCSM4**	RCP4.5	80943.3	12.2	24005.7	3.6	90798.2	13.7	26124.0	3.9
	RCP8.5	103845.1	15.7	27346.5	4.1	119696.7	18.0	36397.1	5.5
**HadGEM2-CC**	RCP4.5	111110.2	16.8	30215.2	4.6	122311.2	18.4	34571.2	5.2
	RCP8.5	123552.1	18.6	34912.2	5.3	136008.0	20.5	45316.3	6.8
**MPI-ESM-LR**	RCP4.5	62044.3	9.4	25608.8	3.9	65914.8	9.9	28708.4	4.3
	RCP8.5	65947.8	9.9	29920.6	4.5	80184.8	12.1	37000.1	5.6
**MRI-CGCM3**	RCP4.5	76174.8	11.5	22208.5	3.3	82143.1	12.4	25428.1	3.8
	RCP8.5	91936.0	13.9	26152.2	3.9	115506.7	17.4	35181.5	5.3

The whole projection area is 663264.7 × 10^3^ ha. The suitable land under current conditions is 56691.3 × 10^3^ ha. Values are expressed in 10^3^ ha.

The model projection under current conditions showed two main potential areas suitable for white mustard cultivation, one on the southwest coast of the Mediterranean (including the southern Iberian Peninsula, northern Morocco and Algeria, and Tunisia) and the other in the eastern Mediterranean (Fig 2). Ensemble projections for the future distributions (2050 and 2080) suggest a displacement of the potential suitable area to the north. In 2050, the expansion would be substantial in northern Algeria, northeastern Morocco, Mediterranean Europe, the Bengasi coast, the north coast of Egypt, the Sinai Peninsula, the Near East coast, and western Georgia (Fig 3A and 3B). The area decreases in the interior part of the Gulf of Gabes and the confluence of Iraq, Jordan, and Saudi Arabia (Fig 3A and 3B). In 2080, the most remarkable change in distribution is the consolidation of the Mediterranean region as the optimum climatic area for the cultivation of the species (Fig 3C and 3D), increasing the suitable area to the north, especially considering the more drastic GCMs and emission scenarios. We also observed increases in the Bengasi coast, the north coast of Egypt, the Sinai Peninsula, the Near East coast, and west of Georgia, but to a lesser extent than those in 2050. The extent of suitable areas are expected to decrease in the interior part of the Gulf of Gabes, the north coast of Algeria, and the areas of confluence of Iraq, Jordan, and Saudi Arabia (Fig 3C and 3D).

### Rapeseed crop suitability index

**Fig 4:**
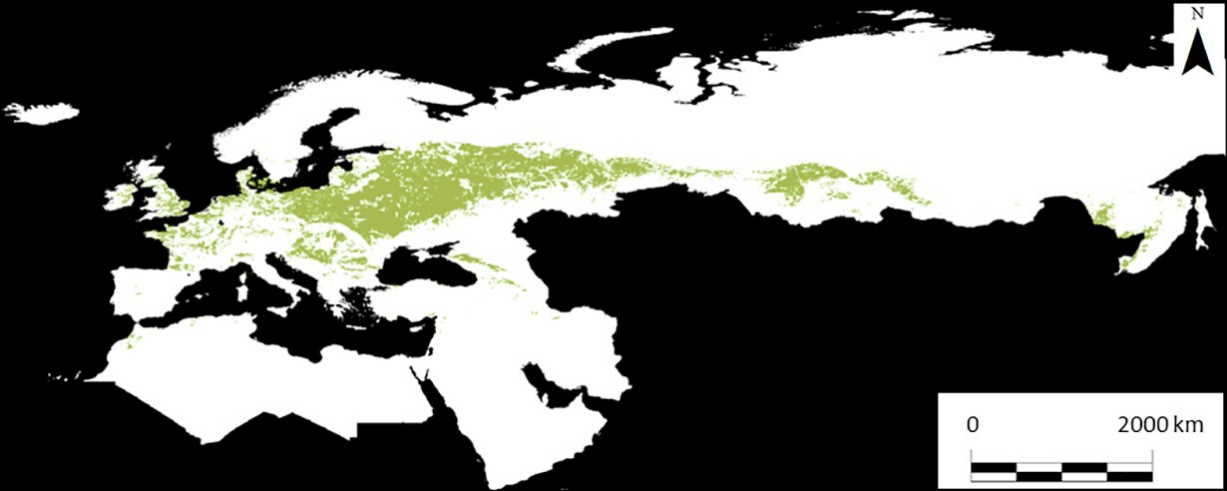
Rapeseed crop suitability model (according to GAEZ) under current conditions. The colored areas show the areas in which rapeseed is currently grown.

**Fig 5:**
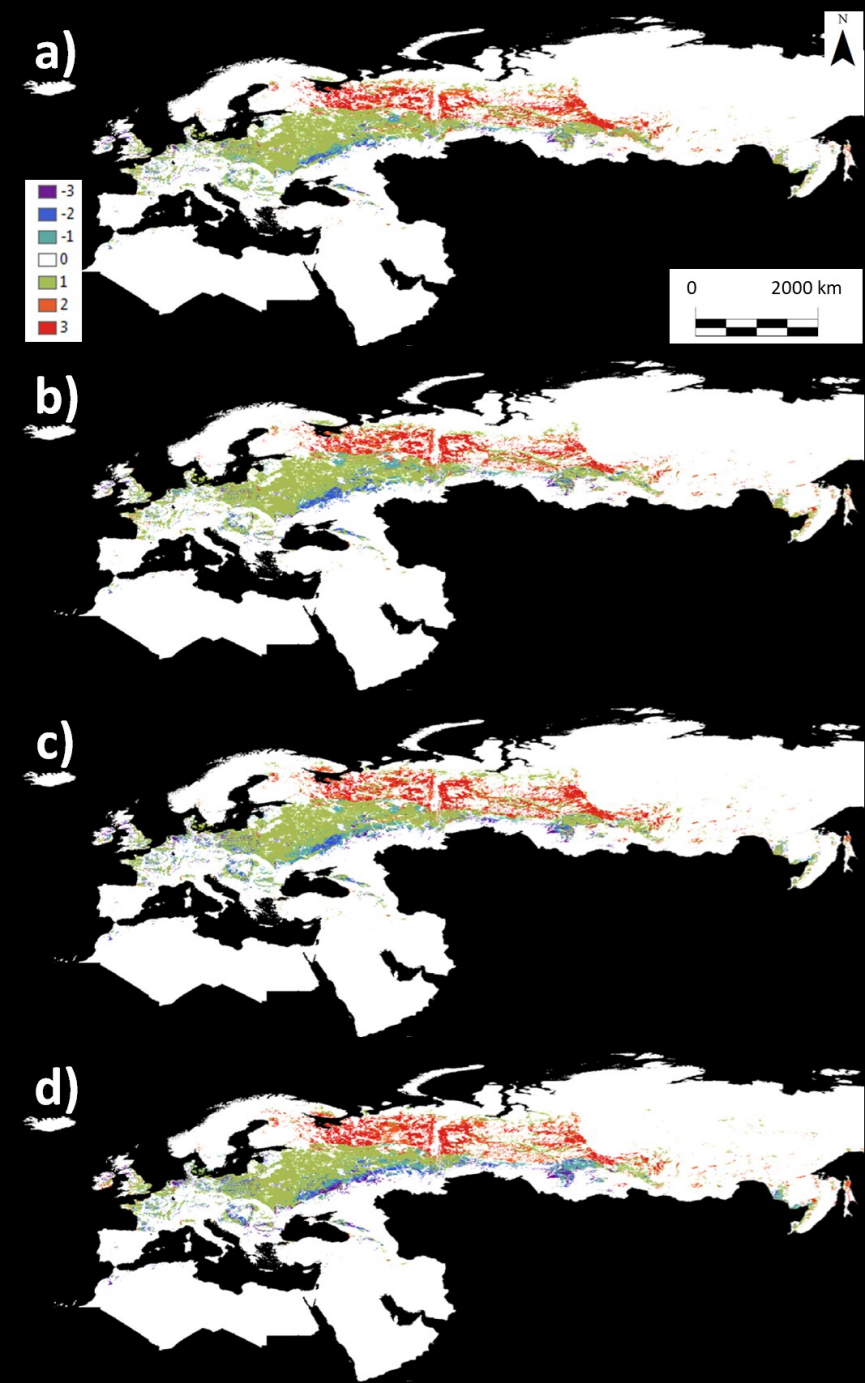
Ensemble forecast maps of rapeseed suitability based on three GCMs for combinations of year and greenhouse gas emission scenarios. a) 2050 and B2 emission scenario, b) 2050 and A2 emission scenario, c) 2080 and B2 emission scenario, and d) 2080 and A2 emission scenario. Suitability is represented on a scale from -3 to 3, where negative values indicate the number of GCMs predicting that a currently suitable pixel will become unsuitable for the species, 0 indicates that the species is not present currently or in the future according to all GCMs, and positive values indicate the number of GCMs predicting that a currently suitable pixel will remain suitable for the cultivation of the species.

The current model of the crop suitability index, using GAEZ, indicated that in our projection area there are 531607.1 × 10^3^ ha of suitable land to grow rapeseed (Fig 4). The four ensemble projections for 2050 and 2080 under A2 and B2 emissions scenarios indicated that this area would increase to between 646658 × 10^3^ and 737450 × 10^3^ ha in 2050 and between 582603 × 10^3^ ha and 734252 × 10^3^ ha in 2080 (Fig 5; Table 3), i.e., a 13.1% increase in 2050 and 19.8% in 2080 of currently suitable land. We observed increases in suitable areas almost exclusively in the northeast, mainly in currently unsuitable areas of Siberia. By contrast, Central and Eastern Europe will lose suitable areas for rapeseed cultivation under future climate scenarios. In particular, this decrease in southern areas would be around 70000 × 10^3^ ha in 2050 and 105000 × 10^3^ ha in 2080 (Fig 5). When we consider only EU countries (with 140964.4 × 10^3^ ha of currently suitable land to grow rapeseed), the four ensemble forecasts show a net reduction in suitable areas of an average of 16000 × 10^3^ ha in 2050 and 26400 × 10^3^ ha in 2080, or 11.3% of currently suitable land in EU countries in 2050 and 18.7% in 2080.

**Table 3:** Extent of suitable land to grow rapeseed under different climate change scenarios.

General circulation model	Greenhouse gas emission scenario	Year	Whole suitable land	Area lost relative to 1996 in the projection area	Area gained relative to 1996 in the projection area	Absolute gain/loss in the projection area	EU countries suitable land	Area lost relative to 1996 in EU countries	Area gained relative to 1996 in EU countries	Absolute gain/loss in EU countries
**CSIRO Mk2**	A2	2050	646657.9	101382.2	216433.0	115050.7 (21.6)	128959.4	19979.8	7974.8	-12005.0 (-8.5)
		2080	582602.7	150294.0	201289.6	50995.5 (9.6)	130194.2	23787.1	13016.9	-10770.2 (-7.6)
	B2	2050	698013.6	83100.3	249506.8	166406.4 (31.3)	128547.8	20022.6	7606.0	-12416.6 (-8.8)
		2080	640192.4	112958.5	221543.7	108585.2 (20.4)	122596.8	25056.2	6688.5	-18367.6 (-13.0)
**Hadley CM3**	A2	2050	681558.2	78032.5	227983.5	149951.0 (28.2)	128307.7	20082.7	7426.0	-12656.7 (-9.0)
		2080	672777.4	120890.4	262060.6	141170.2 (26.6)	109665.7	38647.5	7348.8	-31298.8 (-22.2)
	B2	2050	682775.8	66945.0	218113.7	151168.7 (28.4)	122888.3	22586.6	4510.5	-18076.1 (-12.8)
		2080	680443.4	102402.7	251238.9	148836.3 (28.0)	112821.3	34402.9	6259.8	-28143.2 (-20.0)
**MPI ECHAM4**	A2	2050	737450.0	36649.6	235015.0	198365.5 (37.3)	132483.8	21257.4	6911.5	-14346.0 (-10.2)
		2080	734251.5	80990.9	275677.7	194686.8 (36.6)	111449.3	44247.0	9158.1	-35088.9 (-24.9)
	B2	2050	718507.8	54074.0	240974.7	186900.7 (35.2)	114381.9	32284.9	5702.4	-26582.5 (-18.9)
		2080	728446.3	63137.7	259976.9	196839.1 (37.0)	105961.3	41897.5	6894.3	-35003.2 (-24.8)

Suitable land in 1996 for the whole projection area was 531607.1 × 10^3^ ha and 140964.4 × 10^3^ ha for EU countries only. In parentheses, the percentages (absolute gain or loss) relative to 1996 are shown. Values are expressed in 10^3^ ha.

## Discussion

The shift in the distribution of suitable areas for economically important crops is an enormous concern in relation to climate change [11, 29, 43]. In this study, we compared the current potential areas of cultivation in Europe and their predicted shifts in relation to climate change for two common mustards with economic value owing to their seed oil contents. We found that, while the current potential areas of cultivation are segregated, allowing for the complementary use of both species in different regions, future areas for rapeseed cultivation will retract considerably in Europe and areas for white mustard will expand in the northern Mediterranean Basin.

Recent studies of key agronomical traits in *S*. *alba*, as seed production, lipid content, and fatty acidic composition, confirmed the potential for biofuel and industrial lubricant production using white mustard (i.e., [25, 44, 72]). These studies showed that the lipid content of white mustard seeds is commonly above 25% of dry matter, reaching 41% [26], and the acid composition is analogous to those of other mustards, such as canola and soybean, used for biodiesel production. Likewise, the feasibility of the trans-esterification of polysaturated fatty acids to methyl ester for biodiesel has been demonstrated [26, 39]. The results of this study combined with the potential for biodiesel production using white mustard suggest that more agronomic, ecological, and environmental research focused on this species is needed to promote its use as an alternative to rapeseed for biofuel production in Europe.

### Do currently suitable areas for rapeseed and white mustard overlap?

We only sampled a portion of the entire distribution of white mustard; accordingly, our model may underestimate the suitable area for the species. However, the model prediction under the current climate closely matched the known native distribution of the species according to the Atlas of the European Flora (see [73–74]) and was concordant with the general climatic preferences that characterize the habitats of the species. White mustard is widely distributed around the Mediterranean Basin, showing two main potential areas of distribution, one on the southwestern coast of the Mediterranean and another in the eastern Mediterranean. Both areas are characterized by high aridity (high temperatures and low precipitation), especially during the summer. The rapeseed distribution model developed using GAEZ indicated that the current rapeseed distribution mainly includes Central and Eastern Europe (see Fig 4). Distribution models under current conditions show a lack of overlap in suitable areas for the cultivation of these two crop species, which makes the complementary cultivation of both species at the continental level feasible for biofuel production (see Fig 2 and Fig 4). The production of white mustard seeds is of particular interest, since it could be grown throughout most of the Mediterranean Basin.

White mustard is native to the Mediterranean region, where it is adapted to the climatic conditions, and it has great potential as a native source for biodiesel production. Because it contains much higher erucic acid levels than rapeseed, its oil is unsuitable for human consumption [26]. Thus, these two crops do not overlap in space or cultivation purpose, since white mustard crops are not oriented for the agro-alimentary oil market and are almost exclusively used for biofuel and mustard production.

### Will the distribution of suitable areas for these species expand or retract in Europe during the 21st century under different climate change scenarios?

Climate change could affect agriculture in different ways in different parts of the world [6]. How climate change affects crops depends on current climatic and soil conditions, the direction of change, the spatial distribution of crops, and the availability of resources and infrastructure to cope with changes [12, 45]. The vulnerability of European agroecosystems to climatic conditions, which is the focus of this study, will increase as the temperature rises and precipitation falls [45]. Intensive crop systems in Northwestern Europe have low sensitivity to climate change because farmers have the resources to adapt to new climatic conditions by crop management [45, 75–76].

We compared the potential areas of cultivation for rapeseed and white mustard to determine the extent to which areas unsuitable for rapeseed in the future may be used for white mustard fuel production. The white mustard future projection models show an increase in suitable areas in Southern Europe, with the potential suitable area moving northward (See Fig 3 and Table 2), avoiding the progressively adverse conditions in the southern parts of the Mediterranean. This would increase the suitable area of white mustard for biofuel production in Europe. Rapeseed future projection models also indicate an increase in suitable areas for cultivation. However, this increase in the suitable surface occurs to the northeast, moving mainly towards presently inhospitable areas of Siberia. By contrast, suitable areas in Central and Eastern Europe, where rapeseed is currently harvested, would decrease. In other words, current suitable areas for rapeseed crops will retract in Europe. This result agrees with those of Donatelli et al. [11] for simulations of three major crops in Central and Eastern Europe; they showed that increasingly dry weather in 2030 would make it difficult to maintain rapeseed crops. Donatelli et al. [11] showed that water stress might be a concern in parts of France, Germany, and the UK, where the 2020 projection anticipates a reduction of 5–30% in rapeseed yield, with further reductions in the 2030 projection. In another study [23], this increase in aridity was predicted to reduce the rapeseed oil concentration and seed yield. Nevertheless, this decrease in suitable areas for rapeseed in EU countries (averages of 16014 × 10^3^ ha in 2050 and 26445 × 10^3^ ha in 2080) would be clearly offset by the nearly 48 × 10^6^ net hectares (average among different GCMs and emission scenarios for 2080) of climatically optimum area for white mustard predicted for the Mediterranean region. Thus, white mustard crops would not displace the rapeseed crops but could be planted in areas no longer suitable for rapeseed, enabling an enhanced total suitable area for bioenergy crops in Europe.

### Should European countries focus on the research and development of white mustard oilseeds as an alternative to rapeseed for biofuel production in Europe?

The use of genetically improved oleaginous seeds is at the forefront of current technologies for biodiesel production [18, 25–26]. However, several factors limit the development of energy crops for biodiesel production in Southern Europe. There are ecological constraints to the establishment of oilseed crops from the most commonly used oilseed species (i.e., rapeseed, soybeans, and sunflowers). Recent studies based on current and future ecological niche models have predicted that the distribution of temperate crop species, such as oilseeds, cereals, starches, and solid biofuel crops (pruning, pellets, etc.), will increase in Northern Europe in the coming decades due to an increase in temperatures but will decrease in Southern Europe (Spain, Portugal, Italy, Greece, and the south of France) due to increased aridity [43]. All models indicate that the Iberian Peninsula will be especially vulnerable to climate change, predicting that the cultivation of crop species adapted to temperate climates will decrease dramatically [76]. Thus, bioenergy crops in Southern Europe, and particularly in the Iberian Peninsula, may be seriously compromised unless action is taken to mitigate the effects of climate change [43] and/or to develop crops that are preadapted to drought and aridity.

The recent expansion and intensification of bioenergy crops have raised environmental concerns regarding their real impacts on air, water, and soil quality and their direct and indirect effects on wildlife and biodiversity as well as the spread of genes and plants into native communities [77]. Soil erosion, degradation of water resources, or the conversion of land from natural vegetation to energy cropland could affect natural ecosystems and reduce available natural habitats [77]. It may also generate conflicts related to the multi-functional character of the land, since land dedicated to energy crops is not committed to food, timber, fiber production, cattle raising, or other purposes [78]. Thus, energy production in territories already dedicated to agriculture, which is based on plants not suitable for agro-food use and native to these territories, has been proposed as a way of mitigating the negative consequences on biodiversity and the rise in raw food matter [79]. The cultivation of a bioenergy crop species native to the Mediterranean Basin as white mustard, with few ecological requirements (a weed), is particularly interesting to complement other non-annual local crops. Crops can be grown without compromising land for other purposes. In particular, white mustard could be grown within stable croplands, such as olive or almonds orchards, in Southern Europe (Spain, Italy, Portugal, Greece, and southern France), where the species grows extensively as a native weed. It could be intercropped as a managed ground cover between rows of trees, allowing farmers to cultivate two different crops simultaneously (fruits and white mustard oilseed). Beyond providing additional yield, white mustard cultivation would have ecological, environmental, and agronomic benefits. It could serve as a ground-cover herb (native to the region) to decrease soil loss and increase water infiltration and nutrient retention. The species also has well-known bio-fumigant properties, since it contains glucosinolates, which control pathogens, weeds, insects, and nematodes [80]. Adequately managed, it may further contribute to the maintenance of biodiversity and ecosystem function in these agroecosystems. However, towards Central Europe, the cultivation of white mustard would replace in the future to rapeseed in those areas that due to climate change would no longer climatically suitable. Hence, in Central Europe, white mustard should be planted in a relatively similar manner to current rapeseed cultivation to sustain the biofuel industry.

Given the European deficit in gas and petroleum and the strong dependence on external fuel for automotive vehicles, agriculture, and industry, the development of alternative biofuel crops in Europe should be a priority for the energy policy of the European Commission, especially in the context of the strong projected reduction in suitable areas for cultivation of conventional energy crops. In particular, it is strategic for Southern Europe to develop biofuels from its own crops, adapted to Mediterranean environmental conditions and optimized for energy production. This is possible from native species of the Mediterranean region with high lipid contents in their seeds, such as white mustard, which can likely be genetically improved for this purpose, as demonstrated in sister taxa. According to Chalhoub et al. [81], wild oilseed rape does not exist; this crop appears to have emerged as a hybrid due to human agricultural practices, and its subsequent evolution was influenced humans. It would be interesting to identify genetic lines of white mustard with high lipid contents and greater seed production and to optimize its cultivation to assure crop profitability. The competitive capacity or herbicide resistance must be addressed, since these traits are essential for the implementation of integrated and sustainable programs for the management of the flora associated with crops [82].

In addition to agronomic aspects, it is important to account for the environmental impact of new crop species, especially for plants that are considered weeds. The distributions of many weeds have expanded or are predicted to expand in response to climate change [83]. It is probable that the extreme precipitation caused by climate change could favor competition between invasive weeds and crops, thus harming crops [84]. In addition, an increase in temperature will favor the expansion of weeds towards higher latitudes [83], affecting crops and native plants, thereby generating important agronomic problems and impacts in native plant communities. Therefore, detailed agronomical, economical, and ecological studies are necessary when trying to grow a new species in a new area (especially if the species is considered a weed, as it is the case of white mustard), to deal with the agronomic and ecological problems that this could entail.

## Conclusions

Our model forecast that climate change will cause the loss of thousands of hectares of suitable land for bioenergy production using rapeseed in Europe. A candidate crop to replace rapeseed is white mustard, which is adapted to Mediterranean environmental conditions. According to our models, its suitable area for cultivation will expand throughout the Mediterranean Basin and Central Europe. It has good potential as a biofuel crop and could be genetically improved. Moreover, our model clearly shows that the suitable areas for rapeseed and white mustard do not overlap in the present or in the future. Therefore, white mustard could replace rapeseed for biofuel production in areas that become unsuitable for rapeseed in the near future.

The results of this work advocate improving the knowledge and development of white mustard oilseeds as a potential substitute for rapeseed crops in areas where climate change is expected to compromise their cultivation.

## Supporting information

S1 Table ID of the samples, geographical coordinates of the samples and presence/absence of the species involved in this work. (XLS)
